# Anesthetic Considerations in Sturge-Weber Syndrome: A Case Report

**DOI:** 10.7759/cureus.106331

**Published:** 2026-04-02

**Authors:** Neslida Kodra, Karina Escalona, Peter Hsin, Gerald Rosen, Jonathan Tuman, Miguel E Perez-Viloria

**Affiliations:** 1 Anesthesiology, Mount Sinai Medical Center, Miami Beach, USA; 2 Anesthesiology, Florida International University, Herbert Wertheim College of Medicine, Miami, USA

**Keywords:** airway vascular malformations, anesthetic management, difficult airway, intracranial pressure, intraocular pressure, neurocutaneous disorder, seizure disorder, sturge-weber syndrome

## Abstract

Sturge-Weber syndrome (SWS) is a rare congenital neurocutaneous disorder presenting unique anesthetic challenges due to extensive vascular malformations. Key considerations include difficult airway management due to extensive facial and oral angiomas, seizure control, and maintaining stable intracranial and intraocular pressures. This case report discusses the perioperative management of a 57-year-old female with SWS undergoing dental surgery under general anesthesia and synthesizes the current literature on anesthetic considerations and management. Preoperative examination revealed a facial port-wine stain covering more than 75% of the face, extensive oral angiomas, macroglossia, and severe lip hypertrophy, raising concerns for difficult airway management and risk of hemorrhage. A rapid sequence induction was performed following upright preoxygenation, and video laryngoscopy provided a grade I view of the glottis. Endotracheal intubation was accomplished atraumatically, and the case proceeded uneventfully. The patient was extubated but experienced mild desaturation in the post-anesthesia care unit, prompting overnight observation before discharge at baseline oxygenation. This case highlights the importance of anticipating and planning for difficult airway management, maintaining vigilance for perioperative seizure control, and implementing strategies to minimize elevations in intracranial and intraocular pressures.

## Introduction

Sturge-Weber syndrome (SWS) is a rare congenital neurocutaneous disorder characterized by cutaneous, ocular, and cerebral vascular malformations. It is caused by sporadic mutations in the *GNAQ* gene and, less commonly, the *GNA11* gene, leading to abnormal blood vessel development, with an incidence of 1 in 20,000 to 50,000 live births [1]. The hallmark port-wine stain, a facial capillary malformation, occurs in over 90% of patients [2]. Neurological involvement is often present due to leptomeningeal angiomas on the pia and arachnoid layers of the brain, which can trigger seizure activity and impair venous drainage, potentially contributing to increased intracranial pressure (ICP) [2,3]. Ocular disease is also common, with up to 70% of patients developing glaucoma as well as choroidal angiomas, which can lead to retinal detachment and vision loss [1]. From an anesthetic standpoint, SWS presents significant challenges due to vascular malformations involving the face, oral cavity, and airway, which increase the risk of bleeding, distortion of anatomy, and difficulty with mask ventilation or intubation [4]. Additionally, these patients may be on long-term antiepileptic therapy, requiring careful perioperative drug selection and seizure prophylaxis. The presence of leptomeningeal and ocular vascular malformations further necessitates strategies to minimize elevations in intracranial and intraocular pressures [4]. Although individual case reports have described anesthetic considerations in patients with SWS, standardized management guidelines remain limited. We present the anesthetic management of a 57-year-old female with SWS undergoing dental surgery and provide a review of key perioperative considerations to highlight practical strategies for optimizing outcomes in this complex patient population.

## Case presentation

**Figure 1:**
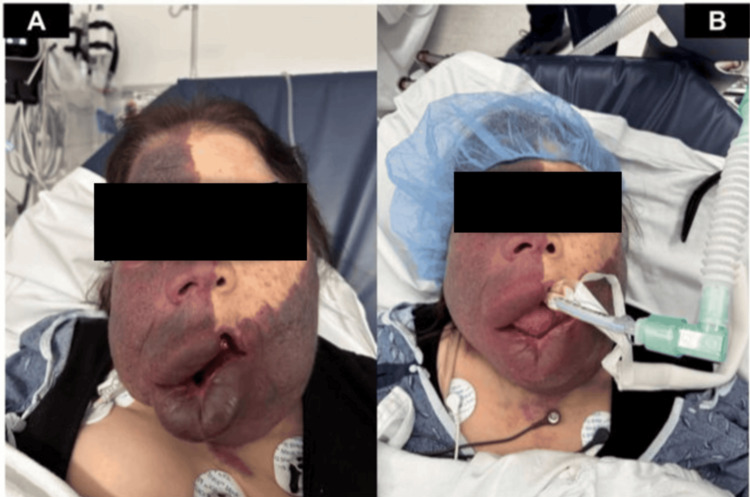
(A) Preoperative photograph demonstrating extensive facial port-wine stain with large angiomas covering the lips and buccal mucosa. (B) Intraoperative view following induction and endotracheal intubation.

A 57-year-old female with a body mass index (BMI) of 27 kg/m² and a history of SWS, glaucoma, and epilepsy presented to our hospital for elective dental surgery under general anesthesia. A physical examination revealed a port-wine stain covering >75% of the face and large oral angiomas involving the lips and buccal mucosa with severe edema and lip protrusion (Figure 1A). Macroglossia and facial hypertrophy were also noted (Figure 1B). Preoperatively, vitals were within normal limits with oxygen saturation at 99-100%. There were clinical signs or a history of increased ICP preoperatively.

**Figure 2:**
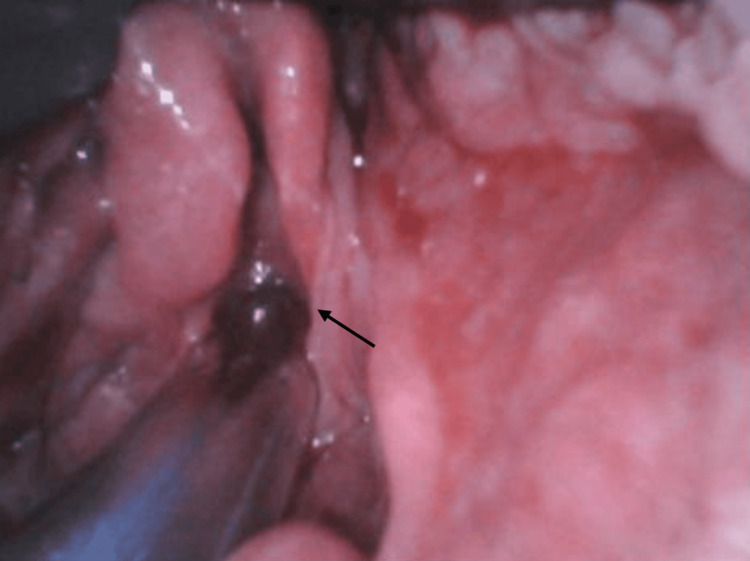
GlideScope-assisted Grade 1 view of the glottis.

Rapid sequence induction (RSI) was planned due to the inability to mask ventilate in the setting of extensive oral vascular involvement and facial edema. Midazolam was administered, and the patient was pre-oxygenated in an upright position with a loose mask. RSI was performed with lidocaine, fentanyl, propofol, and succinylcholine. The patient was then laid supine, and GlideScope-assisted video laryngoscopy was used to visualize the airway. A Grade 1 view of the vocal cords was obtained despite edematous soft tissue and epiglottis (Figure 2). No angiomas were visualized in the pharyngo-larynx, and intubation with a lubricated 7.0 cuffed endotracheal tube (ETT) was successful without bleeding. Care was taken to avoid pressure on facial lesions when securing the tube.

General anesthesia was maintained with sevoflurane at a minimum alveolar concentration (MAC) of 0.8 to 1, supplemented with small boluses of fentanyl and dexmedetomidine. The patient remained hemodynamically stable throughout the procedure with oxygen saturation of 99-100% throughout the procedure. There were no concerns for increased intracranial or intraocular pressures. At the end of the 80-minute procedure, the patient was extubated smoothly but developed mild desaturation with oxygen saturation between 94% and 96% in the post-anesthesia care unit (PACU). Given the risk of potential airway edema, she was admitted for overnight observation and discharged the next morning at baseline oxygenation.

## Discussion

Airway challenges and considerations

Airway management in patients with SWS is complicated by vascular malformations of the lips, buccal mucosa, tongue, gingiva, and, occasionally, the larynx or trachea [4]. The extent of airway involvement may be difficult to assess preoperatively. Although not routinely performed, preoperative magnetic resonance imaging (MRI) may be considered to assess the degree of airway involvement, particularly in cases where significant intraoral or pharyngeal involvement is suspected [2].

Mask ventilation is often difficult due to edema, lip protrusion, and facial asymmetry, which compromise seal and risk angioma rupture. Strategies include preoxygenation with a loose mask or RSI to avoid bag-mask ventilation [4]. Difficult mask ventilation in SWS patients is well documented in the literature, with one case report detailing ventilation using a full-sized face mask that covers the entire face; however, success is variable, as airway seal may still be less than ideal [4].

Laryngoscopy must be approached cautiously as these vascular lesions can cause uncontrolled bleeding, even with minor injury during airway manipulation. Additionally, anesthetic agents induce vasodilation, which can expand these vascular lesions, causing obstruction [2]. Alternative airway management strategies are often necessary to minimize trauma, such as video laryngoscopy or fiberoptic intubation, which offers the additional advantage of visualizing subglottic structures. In select oral and maxillofacial procedures, submental intubation has also been described as a technique to avoid transoral manipulation [4]. Some literature also supports the use of smaller ETTs to minimize pressure on fragile airway tissues [5]. Lubrication of both the tube and blade with jelly can minimize friction, and tubes should be secured without compressing angiomas [6]. Laryngeal mask airways have been safely used in pediatric cases [6].

Postoperatively, patients are at risk for airway edema and vascular engorgement, potentially contributing to hypoxemia and delayed respiratory compromise. While most cases of mild desaturation resolve spontaneously, as it did in this patient, patients with a history of progressive disease may be at higher risk for unexpected airway compromise and respiratory deterioration [5,7]. Given our patient did not have a history of lung disease, smoking, or obesity, her mild desaturation of 94% to 96% was potentially due to transient airway edema that resolved in the 24-hour postoperative period. A summary of the airway management in SWS is presented in Table 1.

**Table 1:** Airway challenges and management specific to Sturge-Weber syndrome. MRI: magnetic resonance imaging; ETT: endotracheal tube; RSI: rapid sequence intubation

Airway considerations	Challenges	Management
Extent of airway involvement	Vascular malformations of the lips, buccal mucosa, tongue, gingiva, and, occasionally, the larynx or trachea	Preoperative MRI is not routinely performed, but potentially useful in cases where significant intraoral or pharyngeal involvement is suspected
	The extent of airway involvement may be difficult to assess preoperatively	
Mask ventilation	Edema, lip protrusion, and facial asymmetry compromise the seal and risk angioma rupture	Preoxygenation with a loose mask or full-sized face mask RSI to avoid bag-mask ventilation
Laryngoscopy	Risk of uncontrolled bleeding during airway manipulation	Video laryngoscopy or fiberoptic intubation (allows for visualization of subglottic structures)
	Anesthetic-induced vasodilation can expand the vascular lesions, causing obstruction	Smaller ETTs and lubricated blades/ETTs can minimize pressure on fragile airway tissue
Delayed respiratory compromise	Postoperatively, patients are at risk for airway edema and vascular engorgement	Most cases of mild desaturation resolve spontaneously. Close postoperative monitoring for hypoxemia and airway compromise

Seizure prevention and management

Seizures are the most common neurologic manifestation in SWS, initially presenting as focal seizures in infancy and progressing to more frequent generalized seizures that can be difficult to control with antiepileptics and are associated with cognitive decline [2,3]. Surgical stress, metabolic changes, and drug interactions can trigger breakthrough seizures. Many antiepileptics can induce hepatic enzymes and alter the metabolism of volatile anesthetics, opioids, and neuromuscular blockers [2]. Twitch monitors can be useful in titrating muscle relaxants, while BIS monitors can help titrate sedation in the setting of potentially altered drug metabolism [2]. Antiepileptics should be continued perioperatively, and restarting oral medications or intravenous alternatives should be considered in prolonged fasting [8]. Standard induction agents such as midazolam and propofol have antiepileptic properties and have been used safely in SWS patients undergoing anesthesia. While studies have shown that sevoflurane has stronger epileptogenic properties compared to isoflurane and nitrous, these epileptiform discharges are typically observed at greater than 1.5 MAC anesthesia. Therefore, sevoflurane is generally considered safe at clinical concentrations in patients with epilepsy [9]. Additionally, factors that can precipitate seizures, such as hypoglycemia, hypotension, hypoxemia, and hyperthermia, should be avoided [2].

Managing intracranial and intraocular pressures

Patients with SWS are at increased risk of developing elevated ICP due to leptomeningeal angiomatosis, which can impair venous drainage of the brain and lead to chronic venous hypertension and progressive cerebral atrophy. Inhalational agents, while generally safe, can increase cerebral blood flow at higher concentrations. Both sevoflurane and nitrous oxide have been safely used for maintenance of anesthesia in SWS patients [6,10]. Propofol and midazolam are often used for their ICP-lowering effects, and ketamine, which was historically avoided due to concerns of increasing ICP, has more recently been shown to be safe in controlled settings [6]. Strategies to minimize ICP include head elevation, normocapnia to mild hypocapnia, and careful fluid administration to prevent excessive volume expansion. Mannitol or hypertonic saline may be considered if there is concern for acute intraoperative ICP elevation [6].

Glaucoma and choroidal hemangiomas also predispose to elevated intraocular pressure (IOP). Several perioperative factors can increase IOP, including positioning (trendelenburg, prone, or lateral positioning), insufflation of gas during laparoscopic or robotic surgeries, endotracheal intubation, coughing and gagging on extubation, and excessive administration of intravenous fluids [11]. Succinylcholine can raise IOP, while inhaled anesthetics and propofol can lower IOP [11]. However, the majority of these factors, with the exception of prolonged positioning, typically only cause short transient spikes in IOP with limited clinical consequence [11]. Therefore, succinylcholine is still routinely used, especially in cases where there is low concern for increased ICP or IOP, such as in this patient.

Considerations for regional anesthesia

Specific guidelines on the use of regional anesthesia in SWS are lacking in the literature. A literature review did not reveal any documented cases of peripheral nerve blocks performed in patients with SWS; however, neuraxial techniques have been described in obstetric patients [12]. While the majority of these cases (8 out of 11) described cesarean sections under general anesthesia (the majority being elective cases), several cases reported the safe use of epidural and spinal anesthesia [12,13]. While neuraxial anesthesia is not contraindicated, caution is advised due to the potential risk of spinal cord angiomas. While leptomeningeal angiomas primarily affect cerebral tissue, spinal hemangiomas have been reported in overlap syndromes (i.e., Klippel-Trenaunay syndrome), creating a theoretical risk [14]. MRI may be useful to assess for spinal involvement, but its use is limited by contrast contraindications during pregnancy. Choosing between general and neuraxial anesthesia in obstetric patients is challenging and lacks consensus. General anesthesia carries the risk of airway difficulty as well as increases in ICP and IOP, particularly during airway maneuvers, while neuraxial anesthesia risks trauma to potential spinal hemangiomas [13].

## Conclusions

Anesthetic management of SWS requires a tailored approach and incorporating strategies for difficult airway management, seizure control, and regulation of ICP and IOP. This case report highlights the importance of a detailed preoperative evaluation, strategic application of advanced airway techniques, including video laryngoscopy and RSI, and vigilant postoperative monitoring. Our literature review further underscores the need for continuation of antiepileptics and cautious selection of anesthetic agents. Awareness of these considerations can optimize perioperative outcomes in this rare but complex patient population.
